# Secondhand smoke exposure among patients with coronary heart disease in a tertiary Grade A hospital in China: A qualitative study of lived experience and sociocultural challenges

**DOI:** 10.18332/tid/225448

**Published:** 2026-09-08

**Authors:** Weimei Yang, Ya Zhao, Lijuan Lu, Xifei He, Xueke Yang

**Affiliations:** 1 Nursing Department of Tongji Hospital, Tongji Medical College, Huazhong University of Science and Technology, Wuhan, China

**Keywords:** coronary heart disease, secondhand smoke, qualitative research, phenomenology

## Abstract

**INTRODUCTION:**

Secondhand smoke (SHS) exposure is a known cardiovascular risk factor, but little is understood about how patients with coronary heart disease (CHD) perceive and manage this exposure in daily life. This study aimed to explore the perceptions, lived experiences, and sociocultural challenges of SHS exposure among Chinese patients with CHD.

**METHODS:**

Face-to-face semi-structured interviews were conducted from December 2023 to April 2024 in the cardiovascular ward of a tertiary Grade A hospital in China, using a descriptive phenomenological approach, among twelve participants. Purposive sampling was used to recruit patients with CHD who reported SHS exposure. Data were analyzed using the Colaizzi seven-step method.

**RESULTS:**

Five themes were identified: 1) Limited and fragmented understanding of SHS; 2) Difficulty avoiding SHS exposure across everyday settings; 3) Aversion to SHS and sociocultural constraints on speaking up; 4) Reliance on smoke-free policies and their enforcement; and 5) Smoke-free homes as an ideal yet difficult-to-achieve goal.

**CONCLUSIONS:**

These qualitative findings suggest that everyday exposure settings, social and relational norms, and the perceived implementation of smoke-free policies shaped SHS exposure among patients with CHD in this study context. Future research with broader and more diverse samples is needed to examine the transferability of these findings and to inform interventions aimed at reducing SHS exposure in high-risk cardiovascular populations.

## INTRODUCTION

Coronary heart disease (CHD) is a leading cause of mortality worldwide and represents a major public health burden^1^. Exposure to secondhand smoke (SHS) is a well-established cardiovascular risk factor^2,3^, and has been linked to adverse cardiovascular outcomes, including acute coronary events and coronary heart disease^4-7^. For patients with CHD, who are already clinically vulnerable, continued SHS exposure may represent an especially important but under-addressed risk in daily life. This issue is particularly relevant in China, where smoking remains common in many households and social settings, and where SHS exposure may persist despite smoke-free policies in some public places^8-11^. Household smoking, social smoking, and culturally embedded norms of hospitality and interpersonal respect may make SHS especially difficult to avoid for patients with CHD.

Much of the existing research on SHS exposure among patients with CHD has used quantitative designs to examine prevalence and associated factors^12,13^. By contrast, prior qualitative studies have focused mainly on other vulnerable groups or household contexts, including pregnant women^14^ and general household smoking practices^8,15,16^. As a result, little is known about how patients with CHD themselves perceive SHS risk, where they encounter SHS in daily life, how they respond when exposure occurs, and how family dynamics, social relationships, and local smoking norms shape their ability to avoid or resist exposure.

To address this gap, this study aimed to explore how patients with CHD in a tertiary Grade A hospital in China perceived SHS exposure, where they encountered it in everyday life, how they responded when exposure occurred, and how sociocultural and relational factors shaped their coping responses. By focusing on lived experience, this study sought to generate contextually grounded evidence relevant to SHS-related risk communication, smoke-free practices, and future intervention development for high-risk cardiovascular populations.

## METHODS

### Setting and participants

This study was conducted in the cardiovascular medical ward of a tertiary Grade A hospital in China. Purposive sampling was used to recruit participants with direct experience of SHS exposure and to capture variation in age, sex, household smoking context, and exposure settings. Inclusion criteria were: 1) aged ≥18 years; 2) clinical diagnosis of CHD; 3) self-reported SHS exposure; 4) ability to communicate effectively; and 5) willingness to participate. Participants were excluded if they had severe comorbidities impairing communication, a diagnosed major psychiatric disorder, a current smoking status, or a critical/unstable health condition.

For recruitment, SHS exposure was defined pragmatically as participants’ self-reported recent or repeated exposure to tobacco smoke from other people in daily life, including at home, at work, or in social/public settings. During screening, participants were asked whether they had experienced SHS exposure in their recent daily lives and whether such exposure occurred repeatedly across common living environments. Because this study aimed to explore lived experience rather than quantify exposure dose, no formal quantitative threshold of frequency, duration, or intensity was imposed.

A descriptive phenomenological approach was adopted because the study aimed to understand how patients with CHD experienced and made sense of SHS exposure in everyday contexts, rather than to generate a formal theory or examine social processes at a more abstract explanatory level^17^. Sampling proceeded concurrently with data collection and analysis until thematic saturation was reached, defined as the point at which subsequent interviews no longer generated substantively new codes, meanings, or thematic insights but instead mainly confirmed the developing thematic structure^18^. A total of 12 eligible patients were approached, and all agreed to participate. Sociodemographic information, smoking history, and CHD history were collected before the interview.

### Data collection

Face-to-face interviews were conducted from December 2023 to April 2024. Each interview lasted approximately 30 minutes and was audio-recorded with participants’ consent. Flexible probing was used during interviews to encourage participants to elaborate on their perceptions, exposure situations, bodily sensations, and coping responses. The interview guide was developed from a literature review and refined after pilot interviews with three patients with CHD. The pilot interviews were conducted to improve question clarity, sequencing, and sensitivity and were not included in the final analysis. The interview guide explored: 1) knowledge and information sources about SHS; 2) perceived health impacts; 3) exposure contexts and coping strategies; 4) perceived facilitators and barriers to reducing exposure; and 5) views on smoke-free homes. Interviews were conducted in Chinese; transcripts were analyzed in Chinese, and illustrative quotations were translated into English for reporting. English translations of the quotations were checked by bilingual researchers to preserve semantic equivalence with the original Chinese transcripts.

### Ethics

This study was conducted in accordance with the principles of the Declaration of Helsinki and was approved by the Ethics Committee of Tongji Hospital, Tongji Medical College, Huazhong University of Science and Technology in in December 2023 (Approval No. TJ-IRB20231235). Written informed consent was obtained from all participants before the interviews. Participants were informed of the study procedures, their right to withdraw, and the measures taken to ensure confidentiality.

### Researcher characteristics and reflexivity

The multidisciplinary research team included nurses specialized in pulmonary and cardiac rehabilitation, and researchers trained in qualitative methods. The primary interviewer had clinical experience in caring for CHD patients but did not provide direct clinical decision-making for enrolled participants. To reduce potential power imbalance, interviews were conducted in a private area of the ward, and participants were reminded that participation or refusal would not affect their care. No prior personal relationship existed between the interviewer and participants before recruitment. Reflexivity was strengthened through field and reflexive notes recorded after each interview, documenting assumptions, emotional responses, and potential leading probes, which were subsequently discussed in regular analytic meetings to support reflexive interpretation.

### Data analysis

Recordings were transcribed using the iFlytek Hearing App for initial transcription, followed by manual verification against the audio by two bilingual researchers. Participant identifiers (P1–P12) were used during analysis. Analysis followed the Colaizzi seven-step method: familiarization, extraction of significant statements, formulation of meanings, clustering into themes, exhaustive description, fundamental structure, and verification^19^. No dedicated qualitative data analysis software was used. Transcripts were coded manually using Microsoft Word and Excel. Two researchers independently coded an initial subset of transcripts, compared coding decisions, and resolved discrepancies through discussion and repeated checking against the original transcripts until consensus was reached. An audit trail was maintained throughout the analysis. Data collection and analysis proceeded iteratively, allowing insights from earlier interviews to inform subsequent interviewing and analytic focus. Member checking was conducted with three participants who were willing and available to be re-contacted and who reflected variation in participant characteristics and exposure experiences. They were invited to comment on whether the preliminary thematic structure adequately reflected their experiences, and their feedback was used to confirm interpretive credibility and refine wording where necessary. This was undertaken to enhance interpretive credibility rather than to re-contact the full sample. To enhance trustworthiness, we used pilot testing of the interview guide, transcript verification, independent coding, reflexive journaling, audit-trail documentation, and member checking. Reporting followed SRQR, and a completed COREQ checklist was prepared^20^ (supplementary file).

## RESULTS

### Participant characteristics

Participant characteristics are presented in Table 1. Of the 12 participants, seven were male, and five were female. Nine participants were ≥60 years. All five female participants reported that their spouses smoked. Among the male participants, most were non-smokers, with one participant reporting a history of smoking.

**Table 1:** Participant characteristics in a descriptive phenomenological study of SHS among patients with CHD conducted in the cardiovascular ward of a tertiary Grade A hospital in China, December 2023 to April 2024 (N=12)

*No.*	*Gender*	*Age (years)*	*Education level*	*Monthly household income (RMB)*	*Family history of CHD*	*Number of hospitalizations*	*Smoking status*	*Spouse smoking status*
1	Male	63	Senior high school	≤2000	Yes	2–3	Never smoker	No
2	Male	70	Junior high school	2000–4000	No	2–3	Never smoker	No
3	Male	55	Junior high school	2000–4000	Yes	1	Former smoker	No
4	Female	50	Senior high school	2000–4000	Yes	2–3	Never smoker	Yes
5	Female	54	Junior high school	≤2000	No	2–3	Never smoker	Yes
6	Male	66	Junior high school	4000–6000	Yes	>3	Never smoker	No
7	Male	60	Senior high school	2000–4000	No	2–3	Never smoker	No
8	Male	62	Junior college	≤2000	No	>3	Never smoker	No
9	Female	65	Senior high school	2000–4000	No	2–3	Never smoker	Yes
10	Male	64	Junior high school	2000–4000	No	2–3	Never smoker	No
11	Female	70	Junior high school	2000–4000	No	2–3	Never smoker	Yes
12	Female	69	Senior high school	4000–6000	No	2–3	Never smoker	Yes

CHD: coronary heart disease. RMB: 1000 Chinese Renminbi about US$150.

### Themes

Analysis of the interview data generated five interrelated themes: 1) Limited and fragmented understanding of SHS; 2) Difficulty avoiding SHS exposure across everyday settings; 3) Aversion to SHS and sociocultural constraints on speaking up; 4) Reliance on smoke-free policies and their enforcement; and 5) Smoke-free homes as an ideal yet difficult-to-achieve goal. Representative quotations are presented in Table 2.

**Table 2:** Themes and representative quotations from a descriptive phenomenological study of SHS among patients with CHD conducted in the cardiovascular ward of a tertiary Grade A hospital in China, December 2023 to April 2024 (N=12)

*Themes*	*Representative quotations*
1. Limited and fragmented understanding of secondhand smoke	*‘It is definitely bad for my health, but I don’t know its specific hazards.’* (P3)
2. Difficulty avoiding SHS exposure across everyday settings	*‘You can’t stop people from smoking in your office … our boss can’t stop it … let alone us.’* (P3)
3. Aversion to SHS and sociocultural constraints on speaking up	*‘We can’t ask people not to smoke. I am ashamed to ask others not to smoke.’* (P2)
4. Reliance on smoke-free policies and their enforcement	*‘It is well managed in Shenzhen. Smoking is not allowed in the factory. In Shenzhen, if someone is found smoking in a restaurant, the restaurant owner will also be fined.’* (P5)
5. Smoke-free homes as an ideal yet difficultto-achieve goal	*‘They don’t smoke in the room, but smoke on the balcony.’* (P12)

### Theme 1: Limited and fragmented understanding of SHS

Participants generally recognized SHS as harmful, but their understanding was often incomplete, experience-based, and lacking in specificity. Many described SHS in simple sensory or experiential terms, such as smoke exhaled by others or smoke that could be smelled in the air, rather than identifying specific biomedical mechanisms or clearly articulating its health consequences. As one participant explained:

*‘Secondhand smoke is what others have smoked, and what you smell belongs to secondhand smoke.’* (P10)

Participants’ awareness of SHS was commonly shaped by fragmented sources, including short videos, television messages, mobile phone content, or stories about illness among relatives and acquaintances. For example, one participant said:

*‘I saw a public video saying secondhand smoke is very harmful* … *and I also saw it on my phone.’* (P1)

while another stated that she had learned from Douyin (Chinese version of TikTok) that:

*‘Secondhand smoke is even more harmful than active smoking.’* (P11)

At the same time, several participants admitted that although they knew SHS was harmful, they could not clearly explain its specific effects. As one participant put it:

*‘It is definitely bad for my health, but I don’t know its specific hazards.’* (P3)

Overall, participants described their understanding of SHS as present but often partial, experience-based, and lacking in specificity.

### Theme 2: Difficulty avoiding SHS exposure across everyday settings

This theme focuses on the settings in which SHS exposure occurred and on the practical constraints that made avoidance difficult. Participants described SHS exposure as a pervasive presence embedded in daily life across the home, workplace, and social settings, often leaving them with little room to avoid exposure without disrupting everyday routines or social participation. At work, exposure was frequently associated with limited power to intervene, particularly in hierarchical or cooperative environments. As one participant explained:

*‘You can’t stop people from smoking in your office, can you? When the boss or employees of other companies come, and they like smoking, our boss can’t stop it* … *let alone our employees.’* (P3)

At home, exposure often came from smoking spouses or family members, and although some participants attempted to restrict indoor smoking, these efforts were only partially successful. For example, one participant said:

*‘My husband smokes … I stop him, but he still smokes … I can’t control him.’* (P4)

while another noted:

*‘If I found him smoking inside, I would push him up onto the balcony.’* (P11)

Social spaces such as tea houses and mahjong parlors were also described as common exposure settings, where avoiding smoke sometimes meant withdrawing from participation altogether. As one participant stated:

*‘If you don’t want to breathe their smoke, you just don’t play cards anymore.’* (P6)

Even brief public encounters, such as passers-by smoking nearby, were mentioned as further reminders of how difficult SHS was to avoid in everyday life. Overall, participants described SHS exposure as occurring repeatedly across multiple everyday settings, with avoidance often constrained by practical and situational factors.

### Theme 3: Aversion to SHS and sociocultural constraints on speaking up

In contrast to the exposure contexts described above, this theme concerns participants’ emotional aversion to SHS and the sociocultural barriers to directly confronting smokers. Although participants strongly disliked SHS, many felt constrained in directly asking others not to smoke. Their responses appeared to vary according to their relationship with the smoker, revealing a clear relational gradient. Participants were generally more willing to remind close family members, but were much more hesitant when dealing with guests, elders, acquaintances, or smokers in public settings. One participant explained:

*‘We can’t force someone to put out a cigarette if we don’t know him very well. You have to respect older people’s habit of smoking.’* (P1)

Another stated:

*‘We can’t ask people not to smoke. I am ashamed to ask others not to smoke* … *To offer someone a cigarette is a courtesy in the countryside.’* (P2)

Similarly, a participant noted that when guests smoked at home:

*‘You can’t ask a guest not to smoke … I’m embarrassed to do so. It’s our culture.’* (P4)

As a result, avoidance became a common coping strategy. Even participants who expressed anger or frustration toward SHS often reported that, in practice, they chose to sit farther away, leave the setting, or remain silent rather than openly challenge smokers. Participants often described a strong aversion to SHS alongside reluctance to speak up, particularly in situations involving politeness, face (i.e. self-image and emotional standing in social interactions) and relational concerns.

### Theme 4: Reliance on smoke-free policies and their enforcement

Participants generally perceived that reducing SHS exposure in public settings could not rely on personal reminders alone, and instead depended largely on formal smoke-free policies and their effective enforcement. Several participants described individual efforts to discourage smoking as socially difficult or practically ineffective, especially in hospitals, workplaces, or other shared spaces. One participant commented:

*‘Now the government has issued a law that does not allow smoking in public places, but there are still many people smoking in the hospital hall. The management in this area needs to be strengthened.’* (P3)

At the same time, participants distinguished clearly between the existence of smoke-free policies and the visible enforcement of those policies. This was reflected in comparisons with places perceived to have stricter management. As one participant noted:

*‘It is well managed in Shenzhen. Smoking is not allowed in the factory. In Shenzhen, if someone is found smoking in a restaurant, the restaurant owner will also be fined.’* (P5)

Participants commonly referred to smoke-free policies as important for protection in public settings while also noting variability in enforcement.

### Theme 5: Smoke-free homes as an ideal yet difficult-to-achieve goal

Most participants regarded smoke-free homes as desirable and beneficial, particularly for health and household well-being. As one participant stated:

*‘The suggestion of smoke-free families is very good. On the one hand, it saves money, and on the other hand, it is good for health.’* (P1)

However, although the idea of a smoke-free home was widely supported in principle, participants often described it as difficult to fully achieve in practice. A recurring pattern was that smoking within the household was managed through compromise rather than complete prohibition. One participant commented:

*‘A smoke-free home would be nice, but it can’t be achieved … I can’t control him.’* (P3)

Others described family members smoking in stairwells, balconies, or just outside the home as a way of reducing, but not eliminating, exposure. For instance, one participant said:

*‘He was afraid to smoke in the house. He smoked outside in the stairwell or on the balcony.’* (P5)

while another reported:

*‘They don’t smoke in the room, but smoke on the balcony.’* (P12)

Participants commonly described smoke-free homes as partially restricted rather than completely smoke-free, with household smoking practices often managed through negotiation and compromise.

## DISCUSSION

This study provides a qualitative account of how patients with CHD in a Chinese tertiary hospital context perceived and navigated SHS exposure in daily life. Rather than depicting SHS as an occasional or incidental exposure, participants described it as a recurrent part of everyday living across the household, workplace, and social environments. These findings suggest that, within this study context, SHS exposure was shaped not only by individual awareness but also by family smoking patterns, social etiquette, and the perceived implementation of smoke-free policies. Participants generally recognized SHS as harmful, but their understanding was often incomplete, particularly with respect to cardiovascular risk. Although many participants described SHS as unpleasant or unhealthy, they were often unable to explain its specific harms. This finding is consistent with prior work suggesting that incomplete SHS knowledge may hinder smoke-free home practices^15,21^. For patients with CHD, such gaps may be especially important because SHS represents a potentially modifiable cardiovascular risk. These findings support the need for more explicit SHS-related cardiovascular risk communication in clinical settings.

Participants described SHS exposure as difficult to avoid across home, workplace, and social settings, which is broadly consistent with prior quantitative studies among patients with CHD^12^. However, this difficulty should be interpreted cautiously as a context-specific qualitative finding rather than a population estimate. A notable feature of the present study was the distinction between exposure occurrence and participants’ response to exposure: although participants strongly disliked SHS, they often felt unable to directly challenge smokers because of politeness, face, age hierarchy, hospitality norms, and relational concerns. This pattern is consistent with previous qualitative work showing that social and interpersonal norms may constrain resistance to smoking^8,16,22^. In this context, avoidance rather than confrontation may function as a socially pragmatic coping strategy^23^. The findings also highlight the importance of family dynamics and household negotiation. Participants generally supported the idea of smoke-free homes, yet most described smoking restrictions as partial and compromise-based, such as smoking on balconies or in stairwells. These accounts suggest that, in this context, a smoke-free home was often understood as a negotiated reduction in indoor smoking rather than a fully smoke-free environment. Similar challenges have been noted in other household-focused studies^15,24,25^. Importantly, such partial restrictions may coexist with continued exposure to SHS or thirdhand smoke^26,27^.

Participants also emphasized the importance of smoke-free policy enforcement in public settings. Rather than referring only to the existence of smoke-free rules, they distinguished between policy presence and visible enforcement. In China, although smoke-free policies have continued to develop^10,11,28,29^, implementation challenges remain^30^. Recent national data indicate that adult smoking prevalence remains substantial, despite a declining trend^9^. For patients with CHD, who are especially vulnerable to SHS, gaps between policy intent and lived protection may remain clinically meaningful. Since the World Health Organization Framework Convention for Tobacco Control (WHO-FCTC) was ratified in 2005, various countries have introduced smoke-free laws to protect people from tobacco smoke exposure in indoor workplaces and public places^29^. The implementation of smoke-free policies may contribute to reduced smoking behavior and lower SHS exposure in some settings^31^. Recent national data indicate that the smoking prevalence among Chinese adults remains high, although a declining trend has been reported^9^. Healthy China 2030 outlines that by 2030, the smoking rate for those aged ≥15 years should decrease to 20%^10^. Smoke-free policies have been implemented in over 20 cities, including Beijing, Shanghai, Shenzhen, and Xi’an^11,28^. However, as participants noted, some local legislation remains insufficiently enforced. Beyond public settings, the findings also highlighted continuing challenges in achieving smoke-free practices within the home. Participants commonly described balcony smoking or smoking outside the room as a compromise intended to reduce direct exposure. However, such arrangements may not eliminate exposure risk, particularly in relation to persistent tobacco residues and incomplete household smoking restrictions^24-27^. These findings may help future intervention development by highlighting the potential relevance of SHS knowledge, misconceptions about partial household smoking restrictions, and socially acceptable approaches to family negotiation.

### Strengths and limitations

Measures against SHS remain a key challenge. Exposure to SHS is closely associated with coronary heart disease. Even brief exposure to SHS can harm the cardiovascular system, which is particularly sensitive to its toxins, much like the effects of active smoking^4^. These findings underscore the potential relevance of SHS exposure in the broader context of secondary prevention for patients with CHD. This study helps clarify how SHS exposure was perceived, negotiated, and managed among participants in this study context. However, this study has several limitations. First, because the findings were based on retrospective self-reports, recall bias and social desirability bias may have influenced participants’ accounts. Second, purposive sampling within a single tertiary Grade A hospital may have introduced selection bias, favoring participants who were more willing or able to discuss SHS exposure. Third, participants were hospitalized patients with CHD, and their clinical vulnerability may have shaped how they perceived and described SHS-related risk. Fourth, although reflexive journaling and team discussions were used to reduce bias, interviewer characteristics and assumptions may still have influenced data collection and interpretation. Finally, the study was conducted in a specific Chinese healthcare and sociocultural context, and the findings should not be assumed to be directly transferable to other populations or settings. In addition, seasonal variation in SHS exposure was not examined in this study and may be explored in future quantitative or longitudinal research. Future research using multi-site, more diverse, and potentially mixed-methods designs is needed to examine the transferability and broader relevance of these findings.

## CONCLUSIONS

This study provides a context-specific, culturally grounded understanding of SHS exposure among patients with CHD in a tertiary Grade A hospital in China. Participants generally recognized SHS as harmful, but their understanding was often incomplete, and their efforts to avoid exposure were shaped by recurring exposure contexts, social and relational norms, and unevenly perceived protection in some public and household settings.

These findings suggest that, in this context, SHS exposure may be understood not simply as an individual behavioral issue, but as a lived experience shaped by the interaction of health vulnerability, family smoking practices, social etiquette, and enforcement environments. The findings may help inform future SHS-related risk communication and family-centered support for patients with CHD in similar settings. Further research with broader and more diverse samples is needed to examine the transferability of these findings and to support intervention development.

## Supplementary Material



## Data Availability

The data supporting this research are available from the authors on reasonable request (subject to ethical approvals and confidentiality agreements).
